# Editorial: Trends in Regulatory Peptides

**DOI:** 10.3389/fendo.2018.00125

**Published:** 2018-03-26

**Authors:** Hubert Vaudry, Marie-Christine Tonon, David Vaudry

**Affiliations:** University of Rouen, Mont-Saint-Aignan, France

**Keywords:** biologically active peptides, G protein-coupled receptors, neuropeptides, gliopeptides, gastrointestinal peptides, peptides and cancer, cardiovascular peptides, antimicrobial peptides

**Editorial on the Research Topic**

**Trends in Regulatory Peptides**

Regulatory peptides play crucial roles in the transfer of information within cells and tissues, between tissues and organs in the body, or between different organisms. They are produced by all species belonging to the different phylums, from bacteria to mammals. They display, by far, the most diverse structures of all signaling molecules. Regulatory peptides exert a broad spectrum of biological effects, acting notably as neurotransmitters, neuromodulators, hormones, pheromones, growth factors, cytokines, toxins, antibiotics, etc. In the animal kingdom, they control all physiological activities including neurophysiological, cardiovascular, gastrointestinal, renal, urogenital, respiratory, and immune functions. Not surprisingly, they are implicated in a number of physiopathological conditions such as pain transmission, Alzheimer’s disease, autism, stroke, tumorigenesis, infertility, diabetes, metabolic disorders, and cardiovascular and gastrointestinal diseases. Thus, a large proportion of drugs target peptide receptors including opiate, vasopressin, oxytocin, somatostatin, gonadotropin-releasing hormone (GnRH), melanocortin, growth hormone, insulin, glucagon, parathyroid hormone, and calcitonin receptors. In addition, a number of pharmacological compounds regulate the production or breakdown of regulatory peptides, e.g., angiotensin-converting enzyme (ACE) inhibitors, dipeptidyl peptidase IV (DPP-IV) inhibitors, and enkephalinase inhibitors. Various peptides are also currently used as vaccines, antibiotics, sweeteners, cosmetic ingredients, and food additives. As a result, biologically active peptides represent a fascinating multidisciplinary research field for chemists, biochemists, physiologists, and pharmacologists with strong potential for novel therapeutic approaches and drug development.

This Research Topic is a compilation of contributions from the Regulatory Peptide meeting (RegPep2016) held in Rouen, Normandy, France that illustrates the diversity of the investigations currently conducted across the world on biologically active peptides.

Many regulatory peptides exert their biological effects through G protein-coupled receptors (GPCRs) which, not surprisingly, are targeted by a large proportion of the drugs currently on the market. Pharmaceutical compounds acting through a given GPCR can differentially activate its downstream signaling pathways (1). This functional selectivity of GPCR agonists is called biased signaling. Gundry et al. described two examples of drug-biased ligands, i.e., dopamine D2 receptor agonists and μ-opioid receptor agonists, which could have practical implications for the treatment of psychiatric disorders and pain. They highlighted the potential limitations in the characterization of biased agonists and provided a general approach to assessing biased agonists that should help the development of this promising new class of drugs.

Fluorescence resonance energy transfer (FRET) and bioluminescence resonance energy transfer (BRET) technologies are widely applied to study GPCR activation and dimerization (2). Using fluorescent biaresenical hairpin binders as acceptors for BRET-based biosensors, Bourque et al. have compared the responses of type 1 angiotensin receptor, prostaglandin F_2α_ receptor, and β_2_-adrenergic receptor to their respective ligands. Their data revealed marked differences in the magnitude and kinetics of the receptor responses to ligand stimulation. This study demonstrates conformational heterogeneity of three GPCRs that belong to the same receptor family, i.e., class A GPCRs.

Neuropeptides represent one of the largest families of regulatory peptides and regulate many physiological functions in the central nervous system (CNS) and in peripheral organs. For instance, corticotropin-releasing factor (CRF) initiates the hormonal response to stress by stimulating the pituitary adrenocortical axis and the sympathetic system. CRF also acts as a neuromodulator within the brain to elicit stress-related behaviors (3). Stengel and Taché reviewed the different mechanisms through which somatostatin signaling suppresses CRF receptor-mediated stress response. Somatostatin acts at the pituitary level to inhibit CRF-induced corticotropin secretion. Concurrently, somatostatin acts centrally to suppress stress-induced activation of the sympathetic system. Somatostatin attenuates the stress response caused by food restriction. Finally, somatostatin agonists counteract the inhibitory effect of CRF on gastrointestinal motor functions. Specific somatostatin receptor agonists can thus be used in preclinical studies to selectively modulate the different components of stress responses.

Somatostatin-producing sensory neurons express the non-selective cation channel TRPA1, a member of the transient receptor potential ankyrin channel family. The activity of TRPA1 can be modulated by inorganic sodium polysulfide (POLY) and dimethyl trisulfide (DMTS), an organic compound naturally occurring in garlic (4). Bátai et al. have compared the effects of POLY and DMTS on sensory neurons in carrageenan-evoked hind paw inflammation. Using genetically modified mice lacking either TRPA1 or the sst4 somatostatin receptor, they show that somatostatin, acting through sst4, is an important mediator of the antihyperalgesic effect of POLY and DMTS.

There is strong evidence that neuropeptide Y (NPY) also attenuates stress responses, anxiety, fear, and autonomic regulations (5). Serova et al. showed that intranasal administration of the Y1R-preferring NPY receptor agonist [Leu^31^Pro^34^]NPY prevents stress-induced depressive-like behavior. Both NPY and [Leu^31^Pro^34^]NPY inhibit the effect of a single prolonged stress on CRF mRNA expression. In contrast, [Leu^31^Pro^34^]NPY enhances, while NPY attenuates, stress-induced glucocorticoid receptor (GR) mRNA expression. The significance of the differential effects of NPY and its Y1R-preferring agonist on CRF and GR expression is discussed.

A number of neuropeptides play key roles in the regulation of glial cell function. Microglial cells, which are responsible for immune surveillance within CNS, express the two types of bradykinin receptors, B_1_R and B_2_R (6). Asraf et al. have examined the role of these receptors in microglial activation. *In vitro*, the B_1_R antagonist R-175, but not the B_2_R antagonist HOE 140, enhances the production of NO and the release of the pro-inflammatory cytokine TNF-α in lipopolysaccharide-stimulated BV2 microglial cells. In transgenic Alzheimer’s disease mice, intranasal administration of R-175, but not HOE 140, augments amyloid burden and causes microglia accumulation in the cortex. These observations support the view that B_1_R modulation may be considered as a potential therapeutic strategy for Alzheimer’s disease.

Both neurons and astroglial cells express hemoglobin (Hb) (7), and the transcription of the Hb gene is enhanced during the preconditioning phase of ischemia (8). Amri et al. showed that Hb exerts a protective effect on hydrogen peroxide-induced oxidative stress and apoptosis in cultured rat astrocytes. The glioprotective activity of Hb is mediated through the PKA, PKC, and MAK signaling pathways. These data suggest that Hb may confer neuroprotection in neurodegenerative diseases.

Activity-dependent neuroprotective protein (ADNP) is a glioprotein which mediates the neuroprotective action of vasoactive intestinal polypeptide (VIP). Mutations of the ADNP gene have been reported in autistic patients (9). In their case report article, Gozes et al. described the clinical phenotype of an 11-year-old girl carrying an ADNP p.Tyr719* mutation, also known as the Helsmoortel–Van der Aa syndrome. The patient exhibits craniofacial asymmetry, autistic stereotypes, global developmental delay, motor skill deficit, and inability to talk. A short bioactive peptide fragment of ADNP, called NAP, represents a possible therapeutic option for patients with the Helsmoortel–Van der Aa syndrome.

Rehfeld has been involved for four decades in the study of the gastrointestinal regulatory peptide cholecystokinin (CCK) (10). In a comprehensive review, Rehfeld summarized the current knowledge regarding CCK and its receptors. CCK is expressed not only in intestinal endocrine I cells but also in brain neurons, peripheral nerves, endocrine cells, cardiocytes, kidney cells, and male germ cells, as well as in the immune system. Consistent with the widespread distribution of CCK and the CCK1 and CCK2 receptors, CCK and related peptides exert a large array of biological effects. Expression of CCK in various types of tumors suggests that CCK could serve as a tumor marker.

Various peptide hormones from the gastrointestinal tract are involved in the control of appetite and energy homeostasis (11), but little is known regarding the possible interplay between these different hormones. Lindqvist et al. have investigated the effect of ghrelin on glucagon-like peptide 1 (GLP-1), glucose-dependent insulinotropic peptide (GIP), and insulin secretion in mice. They show that intravenous injection of ghrelin induces a significant increase in plasma GLP-1 during a glucose tolerance test, but does not affect circulating levels of GIP, insulin, and glucose. *In vitro*, ghrelin inhibits proglucagon mRNA expression in the GLUTag cell line derived from a glucagon-producing enteroendocrine tumor. These data suggest that ghrelin exerts opposite effects on GLP-1 gene transcription and GLP-1 secretion.

Besides their role as nutriments, food-derived peptides can exert multiple regulatory actions. In particular, protein digestion-derived peptides may control appetite through modulation of gut hormone secretion (12). Caron et al. summarized the literature concerning the action of bioactive peptides originating from dietary proteins on the biosynthesis and release of the gastrointestinal hormones CCK and GLP-1. Alimentary peptides can also regulate the activity of the ubiquitous enzyme DPP-IV, which inactivates GLP-1 and GIP by cleaving off their N-terminal residues. Finally, peptides from food proteins can act as agonists of peripheral opioid receptors, whose activation inhibits gastric emptying and causes food intake-induced satiety. Food protein fragments thus represent a cornucopia of regulatory peptides for the control of food intake and glucose homeostasis.

The satiety effect of CCK is mediated by the vagal nerve that conveys the anorexic signal from the gut to the hindbrain (13). Ndjim et al. hypothesized that vagal sensitivity to CCK could be impaired in rats suffering from perinatal malnutrition. They showed that low-protein diet increases food intake after a fasting period, enhances the postprandial plasma CCK level, attenuates the sensitivity to CCK, and reduces the CCK1 receptor level in nodose ganglia. These data support the notion that reduced vagal sensitivity to CCK contributes to food intake disorders in undernourished rats.

Several peptides participate in the regulation of tumor cell proliferation and migration. In particular, VIP inhibits proliferation of small-cell lung carcinoma (SCLC) (14). Since VIP receptors are expressed in bladder carcinoma, Mirsaidi et al. have studied the effect of VIP on bladder cancer cell proliferation *in vivo* and *in vitro*. Intramuscular injection of MB49 bladder cancer cells causes increased mortality in VIP-KO mice as compared to wild-type animals. *In vitro*, VIP reduces the growth of cultured MB49 cells. These data suggest that VIP could be of therapeutic value for the treatment of bladder carcinoma.

Gastrin-releasing peptide (GRP) and neuromedin B are two mammalian counterparts of the frog skin peptide bombesin. All three peptides act through three types of GPCRs designated BB_1_R, BB_2_R, and BRS-3. Numerous cancer cells, including SCLC, express GRP, neuromedin B, and/or their receptor(s), and the peptides act as autocrine factors stimulating tumor growth (15). Moody et al. have synthesized small-molecule bombesin receptor antagonists and tested their ability to antagonize BB_1_R, BB_2_R, and BRS-3 in various cancer cell lines. Two compounds, AM-37 and ST-36, inhibit bombesin agonist (BA)1 binding and (BA)1-induced cancer cell proliferation *in vitro*. These compounds may thus be useful agents for the treatment of BB_1_R-, BB_2_R-, and/or BBS-3-expressing tumor cells.

Gastrin is a peptide hormone produced by the stomach that stimulates gastric acid secretion. Gastrin is also produced by a number of malignant carcinomas called gastrinomas (16). Waldum et al. reviewed the literature on the role of gastrin in the etiology of gastric cancer. Their report points out the predominant role of gastrin in gastric carcinogenesis. It thus appears that gastrin antagonists may prove useful for the prophylaxis of gastric cancer.

Melanoma cells overexpress the melanocortin type 1 receptor (MC1R). Since α-melanocyte-stimulating hormone (α-MSH) is the natural ligand of MC1R, several radiolabeled α-MSH analogs have been proposed for the diagnosis and/or radiopharmaceutical treatment of melanoma. However, high uptake of the labeled peptides in the kidney hampers their clinical applications (17). Bapst and Eberle have designed a new radiolabeled MC1R ligand, [^111^In]-DOTA-Phospho-MSH2-9, with an overall net charge of -1, which exhibits lower kidney uptake and retention. This compound is an attractive novel lead MC1R ligand for the development of clinically relevant melanoma targeting radiopeptides.

The SCLC cell line LU-165 expresses the antidiuretic peptide arginine-vasopressin (AVP) gene. Phenytoin, a voltage-gated sodium (Na_v_) channel antagonist, inhibits AVP release from the isolated rat neurohypophysis and is effective in the treatment of syndrome of inappropriate AVP secretion (18). Ohta et al. showed that phenytoin inhibits AVP mRNA expression in LU-165 cells and suppresses secretion of the C-terminal fragment of pro-AVP from these cells. This study suggests that Na_v_ channels play a key role in AVP expression and secretion in neuroendocrine tumors.

There is clear evidence that GPCRs are implicated in various aspects of tumorigenesis including proliferation, survival, and migration of cancer cells, as well as promotion of angiogenesis (19). Various hormones, acting through GPCRs, play a critical role in progression and metastasis of ovarian cancers. Zhang et al. summarized the literature pertaining to the role of estrogens, GnRH, follicle-stimulating hormone, luteinizing hormone, thyroid-stimulating hormone, and kisspeptin GPCRs in ovarian tumorigenesis, as well as angiotensin II and endothelin GPCRs in neovascularization of ovarian tumors. The diversity of GPCRs regulating growth and metastasis of ovarian tumor cells allows the development of novel chemotherapeutic agents for the benefit of patients with ovarian neoplasms.

Autophagy is a catabolic lysosomal process through which cell compartments are degraded and recycled to maintain energy homeostasis (20). Autophagy plays a key role in cell expansion, chemotactic migration, and invasion (21). Coly et al. summarized the organization and molecular dynamics of the autophagy machinery and elaborated on the implication of chemokine and neuropeptide GPCRs (e.g., CXCR4 and UT, respectively) in the control of autophagosome biogenesis and cancer cell metabolism.

Regulatory peptides exert multiple functions in the cardiovascular system (22). In particular, the vasoactive peptide apelin enhances cardiac contractility and induces the release of vasodilators. The major molecular form of apelin circulating in the plasma is pyroglutamyl apelin 13 ([Pyr^1^]apelin-13). Yang et al. reported that incubation of [Pyr^1^]apelin-13 with recombinant human ACE2 generates the [Pyr^1^]apelin-13_(1-12)_ peptide *in vitro* and that endogenous [Pyr^1^]apelin-13_(1-12)_ is present in the human cardiovascular endothelium. [Pyr^1^]apelin and [Pyr^1^]apelin-13_(1-12)_ bind to human heart with similar affinity, and both peptides induce contraction of the saphenous vein with similar potency. In human volunteers, [Pyr^1^]apelin and [Pyr^1^]apelin-13_(1-12)_ provoke similar dose-dependent increases in forearm blood flow. This study indicates that deletion of the C-terminal phenylalanine residue of [Pyr^1^]apelin-13 by ACE2 does not affect the cardiovascular activity of the peptide.

In plasma, apelin peptides have a short half-life of about 5 min (23). Flahault et al. described the development of metabolically stable and potent apelin analogs that can be used to investigate the cardiovascular and diuretic activities of the native peptide. They provided an extensive and critical look at the physiological effects of apelin on the hydromineral balance and focused on the central and peripheral actions of apelin agonists on cardiorenal functions.

In teleost fish, receptors for the vasoactive neuropeptide urotensin II (UII) are expressed in the brainstem, in the spinal cord, and in the cardiovascular system, suggesting that UII may act both centrally and peripherally to control cardiovascular activity. Consistent with this hypothesis, intracerebroventricular (ICV) or intra-arterial (IA) injection of UII in trout increases blood pressure (BP) (Vanegas et al.). Lancien et al. have studied the effect of UII on the cardiac baroreflex sensitivity (BRS) in unanesthetized trout. They showed that ICV administration of low picomolar doses of UII not only increases BP and heart rate but also reduces BRS, whereas IA administration of UII elevates BP and decreases heart rate without affecting BRS. It thus appears that only the central urotensinergic system is implicated in regulation of BRS.

In addition to its well established vasoactive properties, UII may exert various other biological effects (24). In particular, the genes encoding UII and its receptor UT are expressed in several tumoral cell lines, and UII triggers cancer cell motility. Based on these observations, Castel et al. hypothesized that the UII/UT system could exert chemotactic activities. In support of this hypothesis, they point out the existence of a common proline residue in transmembrane domain 2 (P2.58) shared by UT and chemokine receptors. They also discuss recent studies suggesting that UII may exert pro-inflammatory and pro-migratory effects on tumor cells.

The antimicrobial peptide database (http://aps.unmc.edu/AP) contains currently over 2,850 antimicrobial peptides (AMPs) that generate hope for the treatment of bacterial resistant injections. However, to date, no AMP has led to the development of pharmaceutically useful compounds. Li et al. highlighted the importance of understanding the mechanisms of action of AMPs on the bacterial membrane at the atomic level for the rational design of AMP-derived antibiotics.

The skin, which produces a number of biologically active peptides and expresses their cognate receptors, can be regarded as an authentic neuroimmunoendocrine organ (25, 26). For instance, in the human skin, sensory afferent C-fibers contain the neuropeptides substance P (SP) and calcitonin gene-related peptide (CGRP). N’Diaye et al. described the regulatory actions that SP and CGRP exert on the cutaneous bacterial microflora. This report provides evidence for immunomodulatory functions of SP and CGRP in the maintenance of skin microbiota homeostasis.

Quorum sensing is a chemical communication process by which bacteria regulate gene expression in response to fluctuations in cell population density. Quorum sensing bacteria synthesize different types of auto-inducers: Gram-negative bacteria mainly produce homoserine lactone molecules while Gram-positive bacteria use modified oligopeptides (27). Verbeke et al. described various methods currently available for the identification and measurement of quorum sensing molecules with special emphasis on autoinducer peptides.

The review articles and original research papers gathered in the present e-book illustrate the importance of regulatory peptides in basic research and their huge potential for drug development. We hope that this Research Topic will become a major set of references for all scientists involved in this rapidly expanding field.

## Author Contributions

All the authors have made a substantial, direct, and intellectual contribution to the work and approved it for publication.

## Conflict of Interest Statement

The authors declare that the research was conducted in the absence of any commercial or financial relationship that could be constructed as a potential conflict of interest.
